# Supplementary material for: Assessing diagnostic accuracy of onchocerciasis rapid diagnostic tests using Bayesian latent class models
Source: PLoS Negl Trop Dis. 2026 Oct 7;20(10):e0013954. doi: 10.1371/journal.pntd.0013954 (PMC13660989; doi:10.1371/journal.pntd.0013954)
Supplement: Supplementary file 3 [file pntd.0013954.s006.docx]

**Summary of the Research and Overall Impression:**
This manuscript tackles a critical issue in onchocerciasis elimination: the diagnostic accuracy of Ov16 rapid diagnostic tests (RDTs). The use of Bayesian latent class models is appropriate given the absence of a gold standard, and pooling data from three countries strengthens the analysis. However, several aspects require clarification and improvement.

**Major issues**

1. **Clarity of Methods**
   - Expand the description of the Bayesian latent models. Readers unfamiliar with LCMs may struggle to follow the assumptions and priors used.
   - Justify the choice of priorities for the SD Bioline test (manufacturer vs. field estimates) and discuss how these influence posterior estimates.
2. **Interpretation of Results**
   - The finding that none of the tests met WHO’s specificity threshold needs stronger discussion. What programmatic risks arise from specificity below 99.8%?
   - Explain variability in sensitivity across models (e.g., SD Bioline falling below threshold in Model 2). What does this suggest about reliability across contexts?
3. **Presentation of Data**
   - Tables summarizing posterior estimates are difficult to interpret. Ensure credible intervals are consistently reported.
   - Include graphical summaries (e.g., posterior density plots, forest plots) to make comparisons clearer.
4. **Study Limitations**
   - Discuss potential biases in sampling (e.g., Benin households selected based on child presence).
5. **Geographic Context**
   - I strongly recommend adding a **map of the study areas (districts in Benin, Ghana, Mozambique)**. This would improve clarity, allow readers to appreciate geographic context, and highlight heterogeneity in transmission settings.

**Minor issues**

- Streamline the abstract to focus on the gap addressed.
- Standardize terminology (“Ov16 rapid test” vs. “RDT”).
- Emphasize practical implications for national programs in the author summary.

**Confidential comments for the editors**

The manuscript presents valuable data and uses a robust analytical approach. However, revisions are needed to strengthen methodological clarity, interpretation, and contextual presentation. I recommend major revision; the manuscript has high potential for publication after these issues are addressed.
