# Supplementary material for: Assessing diagnostic accuracy of onchocerciasis rapid diagnostic tests using Bayesian latent class models
Source: PLoS Negl Trop Dis. 2026 Oct 7;20(10):e0013954. doi: 10.1371/journal.pntd.0013954 (PMC13660989; doi:10.1371/journal.pntd.0013954)
Supplement: Supplementary file 4 [file pntd.0013954.s008.docx]

Dear Editor and Reviewers:

Thank you for the opportunity to submit a revised draft of the manuscript "Assessing diagnostic accuracy of onchocerciasis ~~Ov16~~ rapid diagnostic tests ~~for onchocerciasis~~ using Bayesian latent class models" for publication in PLOS NTD. We have updated the manuscript title to reflect that one of the tests–the DDTD RDT–is a multi-antigen test that includes more than just the Ov16 antigen. We are grateful for the reviewers’ careful reading and constructive comments, which have substantially improved the manuscript. Below we provide a point-by-point response, shown in blue. All page and line references refer to the revised manuscript with tracked changes. Where a comment prompted a change, we indicate the location of the change; where we did not make a change, we explain our reasoning.

**Reviewers' Comments & Suggestions to the Authors:**

**Reviewer 1**

1. Expand the description of the Bayesian latent models. Readers unfamiliar with LCMs may struggle to follow the assumptions and priors used.

***Author response:*** *We thank the reviewer for this helpful suggestion. We have added a new Supplementary Material file (S2) that gives the complete specification of the model and a dedicated section stating each modelling assumption together with its justification. The main text now directs readers to S2 for full technical details.*

2. Justify the choice of priorities for the SD Bioline test (manufacturer vs. field estimates) and discuss how these influence posterior estimates.

***Author response:*** *We thank the reviewer for this comment. We assigned an informative prior to SD Bioline, an established assay with independently reported performance data, to ensure identifiability of the latent classes; the two novel RDTs retained noninformative priors so their evaluation would not be influenced by external assumptions. Because manufacturer estimates are typically derived under near ideal conditions, we considered both manufacturer and field-based priors for SD Bioline, alongside a no-prior model, to test the sensitivity of our conclusions to this choice. Exact prior distributions are provided in the supplementary material.*

*The lower SD Bioline sensitivity under Model 2 reflects the downward pull of the field-based prior, not model instability; estimates for the novel RDTs, which used noninformative priors throughout, remained stable across all specifications. We present Models 1–3 as a robustness check on this prior choice rather than as competing results.*

3. The finding that none of the tests met WHO’s specificity threshold needs stronger discussion. What programmatic risks arise from specificity below 99.8%?

***Author response:*** *We appreciate this important suggestion. We have expanded the Discussion to better describe the programmatic implications of specificity estimates below the WHO-recommended threshold, including the potential consequences of false-positive results in low-prevalence elimination settings and their implications for surveillance and decision-making.*

4. Explain variability in sensitivity across models (e.g., SD Bioline falling below threshold in Model 2). What does this suggest about reliability across contexts?

***Author response:*** *We thank the reviewer for this observation. We have expanded the Results and Discussion to explain that the lower SD Bioline sensitivity in Model 2 is a direct consequence of the field-based informative prior (prior mean 74.8%), which pulls the posterior downward relative to Models 1 and 3. The variability therefore reflects alternative prior assumptions for this specific test rather than instability of the analytical method.*

*An informative prior for at least one test helps ensure identifiability of the latent class model and reduces the risk of label-switching between the latent classes; SD Bioline was the natural candidate given available manufacturer and field-based performance data. Sensitivities and specificities for the two novel RDTs, for which no comparable external evidence existed, were assigned noninformative Uniform(0,1) priors to avoid biasing their evaluation.*

*The posterior estimates for the GADx and DDTD tests, which carried noninformative priors in all models, remained essentially unchanged across specifications. We have added text clarifying that the divergence between Model 2 and Models 1/3 for SD Bioline illustrates the sensitivity of conclusions for this test to whether laboratory- or field-derived evidence is considered more credible.*

5. Tables summarizing posterior estimates are difficult to interpret. Ensure credible intervals are consistently reported.

***Author response:*** *Thank you for the suggestion. We reviewed all tables, revised their formatting to improve readability and ensured consistent reporting of posterior median and 95% credible intervals throughout the manuscript and supplementary materials.*

6. Include graphical summaries (e.g., posterior density plots, forest plots) to make comparisons clearer.

***Author response:*** *Thank you for this suggestion. We have developed forest plots for the models into a singular figure. We included the figure caption/legend in the text of the manuscript and uploaded the figure separately (ForestPlots-All.tiff).*

7. Discuss potential biases in sampling (e.g., Benin households selected based on child presence).

***Author response:*** *We appreciate this comment. Our model accounts for country level differences through country specific prevalence parameters. For Benin, this country effect may also conflate the effect of the different population sampling frame used there, households selected based on the presence of a child aged 5 to 9, with true differences in exposure prevalence. However, this does not affect estimation of sensitivity and specificity, which are pooled across countries and are not confounded by country specific sampling design. It only complicates interpretation of the Benin prevalence estimate itself, which is not the focus of the present analysis. We have added text to the Discussion clarifying this point.*

8. I strongly recommend adding a map of the study areas (districts in Benin, Ghana, Mozambique). This would improve clarity, allow readers to appreciate geographic context, and highlight heterogeneity in transmission settings.

***Author response:*** *We thank the reviewer for this helpful suggestion. We agree that additional geographic context improves interpretation of the study settings. Accordingly, we have added a map identifying the study locations and participating districts in Benin, Ghana, and Mozambique (Figure S1) in the supplementary material and refer to this in the main manuscript.*

9. Streamline the abstract to focus on the gap addressed.

***Author response:*** *Thank you for the suggestion. We revised the Abstract to more clearly emphasize the knowledge gap addressed by this study while reducing background information that was not essential to the study objectives.*

10. Standardize terminology (“Ov16 rapid test” vs. “RDT”).

***Author response:*** *Thank you for noting this inconsistency. We reviewed the manuscript and standardized the terminology for the evaluated diagnostic tests throughout to improve clarity and consistency.*

11. Emphasize practical implications for national programs in the author summary.

***Author response:*** *We appreciate this recommendation. We revised the Author Summary to more clearly highlight the practical implications of our findings for onchocerciasis elimination programs, including how these results may inform future diagnostic evaluations and programmatic decision-making. In summary, the GADx RDT performed on finger-prick whole blood met the sensitivity threshold for stop-MDA decisions across all model specifications, removing the need for dried-blood-spot collection and elution required by the current commercial test; however, no evaluated test yet meets the specificity threshold, so stop-MDA decisions should not rest on any of these RDTs alone.*

**Reviewer 2**

1. Harmonization across sites – Please revise the methods to include, for each country:
   a. The target population and sampling frame
   b. How districts/communities/households/individuals were selected (probability vs. Non-probability elements)
   c. Whether any modifications to the sampling occurred mid-collection, and how these changes could affect inference.

***Author response:*** *We thank the reviewer for this suggestion. We have expanded the Methods to more clearly describe the target population, eligibility criteria, and participants selection procedures for each contributing study, using the terminology and descriptions provided in the original study protocols. We also clarify the modification to sampling in Benin, where an additional district was included to achieve the target sample size.*

2. Harmonization across sites – Please clarify the estimand: are you estimating test performance only in the sampled communities, or do you intend these results to generalize to broader districts/countries? If you intend to use pooled estimates, state whether they represent an “average performance” across settings and what assumptions permit that.

***Author response:*** *We thank the reviewer for highlighting the need to clarify the estimand. We have revised the Methods to explicitly state that the objective of this pooled analysis was to estimate the diagnostic sensitivity and specificity of the evaluated Ov16 RDTs using data from the included field studies. The pooled estimates are intended to represent the average diagnostic performance across the study settings under the assumptions of the Bayesian latent class model, rather than nationally representative estimates for the participating countries. We have also clarified this point in the Discussion when interpreting the generalizability of our findings.*

3. Clustering and dependence induced by the design – At a minimum, please justify why clustering can be ignored. Preferably, add one of the following:
   a. a hierarchical model component (e.g., random effects for prevalence by community or site); or
   b. sensitivity analyses stratified by country (and/or district) to demonstrate robustness.

***Author response:*** *We thank the reviewer for raising this point. Our model already stratifies by the coarsest level of clustering in the design: prevalence is estimated separately for each country (Table 1). Sensitivity and specificity, by contrast, are biological properties of each diagnostic test itself, reflecting its ability to detect antibody response given true exposure status, and by definition are independent of prevalence. We therefore treat them as pooled across sites, an assumption also supported by the use of identical test lots and protocols throughout. This is reinforced by an identifiability constraint: with only three tests per participant, and prevalence below 1% in Benin, there are too few truly exposed individuals to support country specific accuracy estimates in that site. Because Se and Sp are defined conditional on latent exposure status, unmodelled clustering primarily widens the true uncertainty around prevalence rather than biasing the accuracy estimates. Pooling test accuracy across sites is standard practice in applications of Bayesian latent class models to multi site data, and we identify hierarchical extensions, such as random effects on prevalence or partial pooling of Se and Sp, as an important direction for future methodological work, now discussed in the Limitations subsection.*

4. Eligibility and age range inconsistencies – Please clarify:
   a. the actual inclusion criteria implemented in each country;
   b. whether there were protocol deviations; and
   c. whether age-specific analyses are needed (or at least a sensitivity analysis excluding participants outside intended eligibility).

***Author response:*** *We thank the reviewer for this comment. We have clarified the eligibility criteria implemented in each contributing study. We have clarified and cross-checked the age ranges, and updates were made verifying that all participants were five and older across all studies; therefore, a sensitivity analysis excluding certain participants is no longer necessary.*

5. Incomplete test panels and complete case analysis – The paper does not adequately describe:
   a. which tests were missing most frequently,
   b. reasons for missing/invalid results (e.g., insufficient sample, invalid strip, logistics),
   c. whether missingness differed by site or participant characteristics.

***Author response:*** *We thank the reviewer for this suggestion. SD Bioline had no invalid results at any site. DDTD had the most invalid results overall (3 in Mozambique, 3 in Benin, 8 in Ghana; 14 total), followed by GADx (2 in Mozambique, 2 in Benin, 1 in Ghana; 5 total). Invalid results were retained in the dataset rather than excluded, since the Bayesian latent class model incorporates whatever test results are available for each participant rather than requiring a complete panel. Reported reasons for invalid results varied by site: in Mozambique, GADx invalid results were attributed to blood discoloration in the results window that obscured the test line, and DDTD invalid results were attributed to the blood transfer device fitting too tightly against the sample port, which could damage the strip; in Benin, invalid results for both tests were attributed to absent control lines in the field. Ghana did not record reasons for its invalid results, and we report the counts without a reason breakdown for that site rather than pursue this retrospectively.*

*We clarified these points in the Data and Statistical Analysis sections.*

6. Please add a participant flow diagram or a structured table (STARD-style) showing denominators by site and by test, and include reasons for missingness. Then, justify the complete-case analysis or, if missingness may be informative, consider a model-based approach to handle missingness or provide sensitivity analyses.

***Author response:*** *We appreciate this suggestion. A formal STARD diagram was not available for this pooled secondary analysis, and we are not able to construct a denominator table broken down by both site and individual test, as attempted-but-missing counts were not captured separately from invalid results at that level of detail. We can report participant-level flow: of 3,057 individuals enrolled across the three sites, 3,014 had a result for at least one test and were included in the analysis. There were 18 participants (15 in Mozambique, 3 in Ghana, none in Benin) who had no result on any test. Reported reasons for this include participant refusal, data entry error, and suspected data-collection app issues in Mozambique, and incomplete dried blood spot collection or participant refusal in Ghana. The Bayesian latent class model incorporated all available test results for each participant rather than requiring a complete panel; only participants with no results at all were excluded. Test-level detail is reported separately for invalid (as opposed to missing) results in our response to the previous comment.*

7. Specimen type and comparability of index tests – Please address this explicitly in the Methods and Discussion:
   a. clarify whether your intended conclusion is “test chemistry performance” or “field workflow performance.”
   b. Provide a rationale for why comparing whole-blood RDTs with a DBS-based test does not introduce differential misclassification that could affect BLCM estimates.

***Author response:*** *We thank the reviewer for raising this important consideration. We have clarified that our objective was evaluation of the diagnostic performance of the tests as implemented under their intended field workflows. We have also expanded the Discussion to acknowledge that differences in specimen type (whole blood versus dried blood spots) may contribute to observed performance differences and represent an important limitation when interpreting comparisons. However, it is important to note that the data utilized for this statistical model were generated through operational research projects, and thus focused primarily on statistical non-inferiority compared to the only existing recommended test (SD Bioline Ov16)- which includes acceptability among field users as well as test performance. The SD Bioline test is recommended by the WHO in the context of being performed on DBS samples in the lab over a period of 24 hours, and from a programmatic standpoint, whole blood from a finger stick sample completed in the field is superior.*

8. Specimen type and comparability of index tests – If feasible, a sensitivity analysis discussing how specimen-related differences could shift sensitivity/specificity would improve interpretability.

***Author response:****We appreciate this suggestion. Because specimen type was inherently linked to the intended workflow of each assay in the contributing studies, whole blood for the two novel tests and dried blood spots for SD Bioline, our data do not permit isolation of specimen-related effects from assay-related effects. We have expanded the Limitations subsection to state this explicitly and to note that our sensitivity and specificity estimates should be interpreted as describing each test's performance under its intended field deployment rather than test chemistry in isolation.*

9. Full model specification and reproducibility - Please provide:
   a. the full likelihood for the 3-test, 8-pattern multinomial data;
   b. whether sensitivities and specificities are assumed constant across countries or allowed to vary (this is crucial when pooling sites);
   c. explicit prior distributions for each parameter (e.g., Beta(a,b) forms), not only prior means.

If code can be shared (even as a supplement), it would substantially strengthen transparency.

***Author response:*** *We thank the reviewer for this helpful suggestion. We have added a new Supplementary Material file (S2) that gives the complete model specification of the model and a dedicated section stating each modelling assumption together with its justification. The main text now directs readers to supplementary material.*

10. Conditional independence assumption – Conditional independence violations can meaningfully bias latent class sensitivity/specificity.
    This is a major methodological vulnerability and needs more than a brief limitation statement. Please add at least one of the following:
    a. a dependent latent class model (e.g., including pairwise covariance terms between tests); or
    b. a sensitivity analysis placing priors on dependence parameters and evaluating how posterior Se/Sp change under plausible dependence.

If you maintain conditional independence, you should justify it with a substantive argument grounded in assay differences and operational separation, and still include quantitative sensitivity checks.

***Author response:*** *We thank the reviewer for raising this important point and agree that conditional independence is a genuine methodological gap that we cannot formally verify with the present data. We have added a fuller discussion to the Discussion section explaining why the assumption may nonetheless be tenable in this setting. The three assays differ in antigen target, platform, and specimen pathway, with the two novel RDTs performed on whole blood at the point of care and the SD Bioline RDT performed on dried blood spots eluted separately in the laboratory, so that shared operational sources of correlated error are limited. We acknowledge a residual source of dependence that this argument cannot remove, since all three tests ultimately detect the same underlying host IgG4 antibody response, and individuals with titres near the detection threshold could plausibly fail multiple tests together. We state the expected direction of bias this would introduce: positive conditional dependence is known to cause latent class models to overstate sensitivity and specificity, so our estimates should be interpreted as upper bounds on true test performance, meaning our central finding, that none of the evaluated tests reaches the WHO specificity threshold, is conservative rather than at risk from this assumption.*

*We also clarify why we have not attempted to estimate dependence directly in the present analysis. With only three tests per participant, and prevalence below 1% in Benin, our model is already only weakly identified, which is why an informative prior was needed for the SD Bioline test to stabilize estimation. Freely estimating a covariance or dependence parameter between tests would add further parameters to an already weakly identified model and could worsen rather than resolve this problem. Establishing the specific biological source of any dependence, for example whether it arises from antibody titres clustering near each test's detection threshold, would require an explicit mechanistic model of the underlying antibody response, which is beyond the scope of the present study.*

11. Pooling across populations: constant vs varying test properties – please justify why test performance is expected to be stable across settings that may differ in epidemiology, co-endemic infections, operational conditions, and specimen handling. If stability is not defensible, consider:
    a. country-specific BLCMs; or
    b. hierarchical partial pooling for Se/Sp.

At a minimum, country-stratified estimates (even if less precise) would help readers assess heterogeneity.

***Author response:*** *Thank you for the comment. The pooled Bayesian latent class model assumes common diagnostic performance across the included study settings while allowing prevalence to vary by country. We have clarified this modeling assumption and have expanded the Discussion to acknowledge that diagnostic performance may vary under different epidemiologic or operational conditions.*

*We would also note that sensitivity and specificity are properties of the diagnostic test itself, defined conditionally on true exposure status, and are independent of prevalence by definition, whereas prevalence is a property of the population and is the parameter we allow to vary by country. With identical protocols and test lots used across sites, we do not see a biological basis for test accuracy to differ by country. More generally, introducing unnecessary complexity into the modelling of sensitivity and specificity, such as allowing them to vary by country without clear justification, is likely to exacerbate the identifiability problems already inherent to latent class models with a limited number of tests.*

12. Prior influence and decision thresholds – Please add:
    a. a clear description of which parameters had informative priors in which models;
    b. a prior sensitivity analysis demonstrating the robustness of conclusions (particularly around key thresholds).

***Author response:*** *We thank the reviewer for this suggestion. The Methods and S1 Text now specify which parameters received informative priors: SD Bioline sensitivity and specificity only, with noninformative Uniform(0,1) priors on all other parameters. Models 1–3 constitute the requested prior sensitivity analysis. GADx and DDTD estimates are stable across all three (GADx 92.0–92.8%, DDTD 86.6–88.4%), so their threshold classification is unaffected by the choice of SD Bioline prior. Only SD Bioline shifts, falling below the 89% threshold under the field prior but not the others, and we now note in the Discussion that this reflects which external evidence is judged more credible rather than model instability. Specificity remains below the 99.8% threshold for all tests under every prior specification.*

13. Results – Please carefully audit:
    a. All entries in Table 1 and any supplemental tables;
    b. Any text describing those values;
    c. Labeling of prevalence (proportion vs. Percentage) to avoid misinterpretation

***Author response:*** *We thank the reviewer for identifying these issues. We carefully reviewed all tables and accompanying text for consistency and made the necessary edits/updates, corrected labeling where needed, and standardized presentation of prevalence estimates and credible intervals throughout the manuscript. We have added a label to the table–Parameters, % (95% CrI)--as well as added a footnote to clarify and avoid misinterpretation of prevalence and all other estimates.*

14. You frame performance against programmatic WHO decision thresholds. To support those objectives more directly, consider reporting decision-relevant quantities rather than only Se/Sp medians: posterior probability that each test meets each threshold (e.g., P(Sp≥0.998∣data)); expected number of false positives per 1,000 tested under low-prevalence scenarios typical of elimination settings (computed from posterior draws); optionally, predictive values over a range of plausible prevalences. These outputs will better connect the statistical model to the operational decisions you reference.

***Author response:*** *We thank the reviewer for this valuable suggestion. We have now computed and report these decision-relevant quantities for all five models: the posterior probability that each test meets the WHO sensitivity (≥89%) and specificity (≥99.8%) thresholds, expected false positives per 1,000 tested at representative low-prevalence scenarios (1%, 5%, 10%), and predictive values (PPV, NPV) across a range of plausible prevalences. Full results are provided in Tables S1a–e (one per model) in S1 Appendix, and we have added a paragraph to the Results section summarizing these findings, including a discussion of heterogeneity across models: GADx's probability of meeting the sensitivity threshold was stable across specifications, SD Bioline's was highly sensitive to the choice of informative prior, DDTD's remained consistently low, no test had an appreciable probability of meeting the specificity threshold under any specification, and DDTD's expected false-positive rate increased notably under the either-line positivity definition (Model 4).*

15. Reframe conclusions to (a) explicitly state they reflect performance in the sampled settings and under the modeling assumptions, and (b) avoid definitive language about “meeting” thresholds unless you provide probability-of-meeting-threshold outputs.

***Author response:*** *We thank the reviewer for this suggestion. We have revised the Abstract, Discussion and Conclusions throughout to replace definitive language about tests "meeting" or "exceeding" thresholds with language describing where posterior estimates and their credible intervals lie relative to the WHO benchmarks. The one exception is our specificity finding, which we retain in stronger form, since the credible intervals for all tests fall entirely below 99.8% in every model; we note in the Limitations that any positive conditional dependence between tests would inflate accuracy estimates, making this conclusion conservative rather than fragile. We have also added a statement to the Conclusions clarifying that our findings reflect performance in the sampled communities under the assumptions of the Bayesian latent class model, and that extending them to nationally representative estimates would require both further methodological development, such as hierarchical models allowing partial pooling across sites, and stronger independent prior information on diagnostic performance than is currently available for these assays.*

16. Add a dedicated “Limitations and robustness checks” subsection, and tie each limitation to (i) direction of potential bias and (ii) what you did (or could do) to assess it.

***Author response:*** *We thank the reviewer for this recommendation. We have added a Limitations section to the Discussion that consolidates the study's principal limitations, addressing conditional independence, prior specification for the SD Bioline test, pooling of test accuracy across countries, specimen type differences, and the operational, non-diagnostic-accuracy design of the contributing studies. Where possible, we state the expected direction of potential bias, and describe our approach and its constraints imposed by both the data and the methods.*

17. The manuscript would advance understanding more convincingly if you sharpen what the dataset uniquely contributes:
    a. multi-country, operational evaluation of Ov16 RDTs in elimination contexts;
    b. comparative performance under realistic field workflows;
    c. evidence that can inform how to design future evaluations and what sample sizes are needed for ultra-high specificity claims.

    To strengthen this, please add:

    a. a clear statement of what is new relative to prior evaluations (e.g., evaluation of the two novel tests at scale, multi-population BLCM, field conditions);
    b. what questions remain unresolved (e.g., performance heterogeneity, dependence, cross-reactivity), and how your data point to next studies.

***Author response:*** *We thank the reviewer for this helpful suggestion. We have added a sentence to the Introduction stating what this study contributes relative to prior work, namely the first evaluation of these two novel Ov16 rapid diagnostic tests alongside the commercially available test across multiple countries using a Bayesian latent class framework. We have also added a paragraph to the Discussion contrasting our approach with two recent evaluations of overlapping sets of these tests, one using SD Bioline as a reference standard in Ghana and one using several parasitological and molecular reference standards in South Sudan. Both of these designs depend on at least one comparator correctly classifying true exposure status, and neither was able to estimate specificity for all three tests. Our latent class framework does not rely on this assumption, and this distinction allowed us to report specificity alongside sensitivity for every test and to identify our central finding, that none of the three tests currently meets the WHO threshold for stopping MDA decisions.*

*The questions that remain unresolved by our data, including heterogeneity of test performance across settings and conditional dependence between tests, are addressed in our responses to the reviewer's earlier comments and in the Limitations subsection of the revised manuscript.*

**Reviewer 3**

1. Data – what samples were collected? Whole blood, dried blood spots? When the authors say multiple Ov16 RDT, how many and which tests were assessed?

***Author response:*** *We thank the reviewer for this helpful comment. We have revised the Methods to more clearly describe the specimen types collected for each diagnostic assay and explicitly identify the three diagnostic tests evaluated in this study in the objective statement found in the Introduction section of the manuscript. We also clarified the terminology used when referring to the evaluated Ov16 rapid diagnostic tests throughout the manuscript.*

2. Index tests – ‘The T1 test line indicates Ov16 and OvOC3261 exposure, and the T2 test line indicates Ov33.3 and OvOC10469 antigens, which are expressed in adult worms and thus indicate established (22, 23)’. Sentence seems to be missing end. Also first these other antigens are mentioned – what is there importance? ‘Ov16 and OvOC3261 exposure’ - exposure to the antigens, is this the right way to phrase this?

***Author response:*** *We appreciate the reviewer for identifying this lack of clarity. We revised the description of the index test to correct the incomplete sentence and streamlined the description of a positive DDTD test result by removing the thorough details about what the test lines represent to avoid confusing readers. Additionally, we have referred to all tests as onchocerciasis RDTs, instead of Ov16 RDTs to be more accurate, reflecting the fact that the DDTD test includes more than the Ov16 antigen.*

3. Results – posterior sensitivity: write out credible interval in full first time it is used before using the abbreviation.

***Author response:*** *We thank the reviewer for noting this issue. We revised the manuscript so that the term "95% credible interval" is written in full as its first appearance in the Results section before subsequently using the abbreviation "95% CrI."*

4. Line numbers would have been useful for the review process.

***Author response:*** *We appreciate this feedback. Line numbers have been added to the revised manuscript submitted for review.*

5. Abstract – methodology and introduction – the authors need to clarify what they mean by pooled data. Is this data from studies the various Ov16 RDT but not specifically assessing the tests at the same time?

***Author response:*** *We thank the reviewer for this helpful suggestion. We revised the Abstract, Introduction, and Methods to more clearly explain that the pooled dataset consisted of participant-level data combined from three coordinated field studies evaluating multiple Ov16 diagnostic tests. We also clarified the rationale for pooling these data within the Bayesian latent class analysis.*

6. Introduction – be clearer about the Ov16 RDT. One paragraph states there are 2 and then the next paragraph says only one is available. And then the final paragraph before the methods introduces 2 novel Ov16 RDT. Might be useful to give each RDT a distinct name.

***Author response:*** *Thank you for identifying this inconsistency. We revised the Introduction to clearly distinguish the commercially available Ov16 rapid diagnostic test (SD BIOLINE Ov16 RDT) from the two novel Ov16 rapid diagnostic tests (DDTD RDT and GADx RDT). We also introduced each assay using consistent terminology throughout the manuscript to improve clarity.*
