# Supplementary material for: Assessing diagnostic accuracy of onchocerciasis rapid diagnostic tests using Bayesian latent class models
Source: PLoS Negl Trop Dis. 2026 Oct 7;20(10):e0013954. doi: 10.1371/journal.pntd.0013954 (PMC13660989; doi:10.1371/journal.pntd.0013954)
Supplement: Peer review history

# Peer review: Decision Letter 0

**DOI:** 10.1371/journal.pntd.0013954.r001

**Authors:** Maria Victoria Periago, Robert Adamu SHEY

11 Mar 2026

PNTD-D-26-00053

Assessing diagnostic accuracy of Ov16 rapid diagnostic tests for onchocerciasis using Bayesian latent class models

PLOS Neglected Tropical Diseases

Dear Dr. Norman,

Thank you for submitting your manuscript to PLOS Neglected Tropical Diseases. After careful consideration, we feel that it has merit but does not fully meet PLOS Neglected Tropical Diseases's publication criteria as it currently stands. Therefore, we invite you to submit a revised version of the manuscript that addresses the points raised during the review process.

Please submit your revised manuscript within by May 10 2026 11:59PM. If you will need more time than this to complete your revisions, please reply to this message or contact the journal office at plosntds@plos.org. When you're ready to submit your revision, log on to https://www.editorialmanager.com/pntd/ and select the 'Submissions Needing Revision' folder to locate your manuscript file.

Please include the following items when submitting your revised manuscript:

* A letter that responds to each point raised by the editor and reviewer(s). You should upload this letter as a separate file labeled 'Response to Reviewers'. This file does not need to include responses to any formatting updates and technical items listed in the 'Journal Requirements' section below.

* A marked-up copy of your manuscript that highlights changes made to the original version. You should upload this as a separate file labeled 'Revised Manuscript with Track Changes'.

* An unmarked version of your revised paper without tracked changes. You should upload this as a separate file labeled 'Manuscript'.

If you would like to make changes to your financial disclosure, competing interests statement, or data availability statement, please make these updates within the submission form at the time of resubmission. Guidelines for resubmitting your figure files are available below the reviewer comments at the end of this letter.

We look forward to receiving your revised manuscript.

Kind regards,

Robert Adamu SHEY, Ph.D.

Guest Editor

PLOS Neglected Tropical Diseases

María Periago

Section Editor

PLOS Neglected Tropical Diseases

Shaden Kamhawi

co-Editor-in-Chief

PLOS Neglected Tropical Diseases

orcid.org/0000-0003-4304-636XX

Paul Brindley

co-Editor-in-Chief

PLOS Neglected Tropical Diseases

orcid.org/0000-0003-1765-0002

**Journal Requirements:**

1) We notice that your supplementary information is included in the manuscript file. Please remove them and upload them with the file type 'Supporting Information'. Please ensure that each Supporting Information file has a legend listed in the manuscript after the references list.

2) Please amend your detailed Financial Disclosure statement. This is published with the article. It must therefore be completed in full sentences and contain the exact wording you wish to be published.

- State the initials, alongside each funding source, of each author to receive each grant. For example: "This work was supported by the National Institutes of Health (####### to AM; ###### to CJ) and the National Science Foundation (###### to AM)."

- State what role the funders took in the study. If the funders had no role in your study, please state: "The funders had no role in study design, data collection and analysis, decision to publish, or preparation of the manuscript.".

If you did not receive any funding for this study, please simply state: u201cThe authors received no specific funding for this work.u201d

**Reviewers' Comments:**

Reviewer's Responses to Questions

**Key Review Criteria Required for Acceptance?**

As you describe the new analyses required for acceptance, please consider the following:

**Methods:**

-Are the objectives of the study clearly articulated with a clear testable hypothesis stated?

-Is the study design appropriate to address the stated objectives?

-Is the population clearly described and appropriate for the hypothesis being tested?

-Is the sample size sufficient to ensure adequate power to address the hypothesis being tested?

-Were correct statistical analysis used to support conclusions?

-Are there concerns about ethical or regulatory requirements being met?

Reviewer #1: (No Response)

Reviewer #2: 1) Study design and sampling

1.1 Harmonization across sites

The three contributing studies appear to use materially different sampling frames and selection mechanisms (e.g., convenience-driven village selection in one setting, household selection contingent on the presence of children in another, and community selection across multiple districts in a third). These differences affect representativeness and comparability and should be described more explicitly and systematically.

Please revise the Methods to include, for each country:

a. the target population and sampling frame.

b. how districts/communities/households/individuals were selected (probability vs non-probability elements);

c. whether any modifications to the sampling plan occurred mid-collection, and how those changes could affect inference.

Please also clarify the estimand: are you estimating test performance only in the sampled communities, or do you intend these results to generalize to broader districts/countries? If you intend to use pooled estimates, state whether they represent an “average performance” across settings and what assumptions permit that.

1.2 Clustering and dependence induced by the design

Because participants are sampled within communities/villages, test outcomes and exposure are likely clustered. Treating all individuals as independent can yield overly narrow uncertainty intervals and may also confound modeling assumptions (e.g., site-level operational factors affecting multiple tests similarly).

At a minimum, please justify why clustering can be ignored. Preferably, add one of the following:

a. a hierarchical model component (e.g., random effects for prevalence by community or site); or

b. sensitivity analyses stratified by country (and/or district) to demonstrate robustness.

2) Eligibility, participant characteristics, and missing data

2.1 Eligibility and age range inconsistencies

The manuscript should reconcile the stated eligibility criteria (e.g., age thresholds) with the reported age distribution (which appears to include ages below those thresholds). This needs to be resolved because test positivity and performance can differ by age and exposure history, and inadvertent inclusion of younger participants could bias estimates.

Please clarify:

a. the actual inclusion criteria implemented in each country;

b. whether there were protocol deviations; and

c. whether age-specific analyses are needed (or at least a sensitivity analysis excluding participants outside intended eligibility).

2.2 Incomplete test panels and complete case analysis

You report a total number of test results and a smaller number of complete observations with all three tests available. The paper does not adequately describe:

a. which tests were missing most frequently,

b. reasons for missing/invalid results (e.g., insufficient sample, invalid strip, logistics),

c. whether missingness differed by site or participant characteristics.

Please add a participant flow diagram or a structured table (STARD-style) showing denominators by site and by test, and include reasons for missingness. Then, justify the complete-case analysis or, if missingness may be informative, consider a model-based approach to handle missingness or provide sensitivity analyses.

3) Specimen type and comparability of index tests

Two tests are conducted on finger-prick whole blood, whereas the comparator test is performed on dried blood spots. This is not only a test comparison but also a workflow/specimen comparison. Specimen type and handling can influence measurement error and the dependence structure between tests.

Please address this explicitly in the Methods and Discussion:

a. clarify whether your intended conclusion is “test chemistry performance” or “field workflow performance.”

b. provide a rationale for why comparing whole-blood RDTs with a DBS-based test does not introduce differential misclassification that could affect BLCM estimates.

If feasible, a sensitivity analysis discussing how specimen-related differences could shift sensitivity/specificity would improve interpretability.

4) Statistical methods: BLCM specification, priors, and assumptions

4.1 Full model specification and reproducibility

The manuscript describes multiple model variants, but the statistical reporting is not sufficient for reproducibility. Please provide:

a. the full likelihood for the 3-test, 8-pattern multinomial data;

b. whether sensitivities and specificities are assumed constant across countries or allowed to vary (this is crucial when pooling sites);

c. explicit prior distributions for each parameter (e.g., Beta(a,b) forms), not only prior means.

If code can be shared (even as a supplement), it would substantially strengthen transparency.

4.2 Conditional independence assumption

All your models assume conditional independence of test results given the latent class. With serology-based tests detecting related antibody signals, conditional dependence is plausible due to shared biology, cross-reactivity, or site-level operational factors. Conditional independence violations can meaningfully bias latent class sensitivity/specificity.

This is a major methodological vulnerability and needs more than a brief limitation statement. Please add at least one of the following:

a. a dependent latent class model (e.g., including pairwise covariance terms between tests); or

b. a sensitivity analysis placing priors on dependence parameters and evaluating how posterior Se/Sp change under plausible dependence.

If you maintain conditional independence, you should justify it with a substantive argument grounded in assay differences and operational separation, and still include quantitative sensitivity checks.

4.3 Pooling across populations: constant vs varying test properties

If you assume common sensitivities/specificities across Mozambique, Ghana, and Benin, please justify why test performance is expected to be stable across settings that may differ in epidemiology, co-endemic infections, operational conditions, and specimen handling. If stability is not defensible, consider:

a. country-specific BLCMs; or

b. hierarchical partial pooling for Se/Sp.

At a minimum, country-stratified estimates (even if less precise) would help readers assess heterogeneity.

4.4 Prior influence and decision thresholds

Some models use informative priors (at least for the comparator test). Because you interpret results against decision thresholds (e.g., sensitivity benchmarks for mapping and stopping decisions and very high specificity requirements), prior choice can meaningfully change decision conclusions.

Please add:

a. a clear description of which parameters had informative priors in which models;

b. a prior sensitivity analysis demonstrating the robustness of conclusions (particularly around key thresholds).

Reviewer #3: Data – what samples were collected? Whole blood, dried blood spots? When the authors say multiple Ov16 RDT, how many and which tests were assessed?

Index tests – ‘The T1 test line indicates Ov16 and OvOC3261 exposure, and the T2 test line indicates Ov33.3 and OvOC10469 antigens, which are expressed in adult worms and thus indicate established (22, 23)’. Sentence seems to be missing end. Also first these other antigens are mentioned – what is there importance? ‘Ov16 and OvOC3261 exposure’ - exposure to the antigens, is this the right way to phrase this?

**********

**Results:**

-Does the analysis presented match the analysis plan?

-Are the results clearly and completely presented?

-Are the figures (Tables, Images) of sufficient quality for clarity?

Reviewer #1: (No Response)

Reviewer #2: Results reporting and internal consistency

1 Check and correct numerical inconsistencies

There appear to be inconsistencies between reported medians and credible intervals (e.g., intervals not containing the point estimate, or intervals that look like they belong to a different parameter such as specificity). This must be corrected, and the narrative must match the tables exactly.

Please carefully audit:

all entries in Table 1 and any supplemental tables;

any text describing those values;

labeling of prevalence (proportion vs percentage) to avoid misinterpretation.

2 Do results address the study’s programmatic objectives?

You frame performance against programmatic WHO decision thresholds. To support those objectives more directly, consider reporting decision-relevant quantities rather than only Se/Sp medians:

posterior probability that each test meets each threshold (e.g., P(Sp≥0.998∣data));

expected number of false positives per 1,000 tested under low-prevalence scenarios typical of elimination settings (computed from posterior draws);

optionally, predictive values over a range of plausible prevalences.

These outputs will better connect the statistical model to the operational decisions you reference.

Reviewer #3: Results – posterior sensitivity: write out credible interval in full first time it is used before using the abbreviation.

**********

**Conclusions:**

-Are the conclusions supported by the data presented?

-Are the limitations of analysis clearly described?

-Do the authors discuss how these data can be helpful to advance our understanding of the topic under study?

-Is public health relevance addressed?

Reviewer #1: (No Response)

Reviewer #2: They are partially supported, but in their current form the conclusions should be tempered. Reframe conclusions to (a) explicitly state they reflect performance in the sampled settings and under the modeling assumptions, and (b) avoid definitive language about “meeting” thresholds unless you provide probability-of-meeting-threshold outputs.

Are the limitations of analysis clearly described? Some are mentioned, but not clearly or sufficiently described for their impact. Add a dedicated “Limitations and robustness checks” subsection, and tie each limitation to (i) direction of potential bias and (ii) what you did (or could do) to assess it.

Do the authors discuss how these data can be helpful to advance our understanding of the topic under study? Only partially. The manuscript would advance understanding more convincingly if you sharpen what the dataset uniquely contributes:

a. multi-country, operational evaluation of Ov16 RDTs in elimination contexts;

b. comparative performance under realistic field workflows;

c. evidence that can inform how to design future evaluations and what sample sizes are needed for ultra-high specificity claims.

To strengthen this, please add:

a. a clear statement of what is new relative to prior evaluations (e.g., evaluation of the two novel tests at scale, multi-population BLCM, field conditions);

b. what questions remain unresolved (e.g., performance heterogeneity, dependence, cross-reactivity), and how your data point to next studies.

Reviewer #3: (No Response)

**********

**Editorial and Data Presentation Modifications?**

Use this section for editorial suggestions as well as relatively minor modifications of existing data that would enhance clarity. If the only modifications needed are minor and/or editorial, you may wish to recommend “Minor Revision” or “Accept”.

Reviewer #1: (No Response)

Reviewer #2: (No Response)

Reviewer #3: (No Response)

**********

**Summary and General Comments:**

Use this section to provide overall comments, discuss strengths/weaknesses of the study, novelty, significance, general execution and scholarship. You may also include additional comments for the author, including concerns about dual publication, research ethics, or publication ethics. If requesting major revision, please articulate the new experiments that are needed.

Reviewer #1: (No Response)

Reviewer #2: (No Response)

Reviewer #3: The manuscript by Norman and colleagues describes the Bayesian laten class model analysis of three rapid diagnostic tests for diagnosis of onchocerciasis. The study analyses 3 available tests that are based on the Ov16 antigen. While Bayesian analysis would typically be used to compare tests focussed on different analytes, it is assumed that each test would be different due to each company proprietary technology. As such the analysis is a crucial piece of work needed for the ongoing efforts to eliminate onchocerciasis. I have minor comments.

Line numbers would have been useful for the review process.

Abstract – methodology and introduction – the authors need to clarify what they mean by pooled data. Is this data from studies the various Ov16 RDT but not specifically assessing the tests at the same time?

Introduction – be clearer about the Ov16 RDT. One paragraph states there are 2 and then the next paragraph says only one is available. And then the final paragraph before the methods introduces 2 novel Ov16 RDT. Might be useful to give each RDT a distinct name.

**********

PLOS authors have the option to publish the peer review history of their article (what does this mean?). If published, this will include your full peer review and any attached files.

If you choose “no”, your identity will remain anonymous but your review may still be made public.

**Do you want your identity to be public for this peer review?**  For information about this choice, including consent withdrawal, please see our Privacy Policy.

Reviewer #1: No

Reviewer #2: **Yes:**  Osama Abdelhay

Reviewer #3: No

[NOTE: If reviewer comments were submitted as an attachment file, they will be attached to this email and accessible via the submission site. Please log into your account, locate the manuscript record, and check for the action link "View Attachments". If this link does not appear, there are no attachment files.]

**Figure resubmission:**  While revising your submission, we strongly recommend that you use PLOS’s NAAS tool (https://ngplosjournals.pagemajik.ai/artanalysis) to test your figure files. NAAS can convert your figure files to the TIFF file type and meet basic requirements (such as print size, resolution), or provide you with a report on issues that do not meet our requirements and that NAAS cannot fix.

After uploading your figures to PLOS’s NAAS tool - https://ngplosjournals.pagemajik.ai/artanalysis, NAAS will process the files provided and display the results in the "Uploaded Files" section of the page as the processing is complete. If the uploaded figures meet our requirements (or NAAS is able to fix the files to meet our requirements), the figure will be marked as "fixed" above. If NAAS is unable to fix the files, a red "failed" label will appear above. When NAAS has confirmed that the figure files meet our requirements, please download the file via the download option, and include these NAAS processed figure files when submitting your revised manuscript.

**Reproducibility:**

To enhance the reproducibility of your results, we recommend that authors of applicable studies deposit laboratory protocols in protocols.io, where a protocol can be assigned its own identifier (DOI) such that it can be cited independently in the future. Additionally, PLOS ONE offers an option to publish peer-reviewed clinical study protocols. Read more information on sharing protocols at https://plos.org/protocols?utm_medium=editorial-email&utm_source=authorletters&utm_campaign=protocols

Attachment Submitted filename: My Comments to the Authors.docx

# Author comment: Author response to Decision Letter 1

**DOI:** 10.1371/journal.pntd.0013954.r002

3 Sep 2026

Attachment Submitted filename: Response to Reviewers.docx

# Peer review: Decision Letter 1

**DOI:** 10.1371/journal.pntd.0013954.r003

**Authors:** Maria Victoria Periago, Robert Adamu SHEY

28 Sep 2026

Dear Ms. Norman,

We are pleased to inform you that your manuscript 'Assessing diagnostic accuracy of onchocerciasis rapid diagnostic tests using Bayesian latent class models' has been provisionally accepted for publication in PLOS Neglected Tropical Diseases.

Before your manuscript can be formally accepted you will need to complete some formatting changes, which you will receive in a follow up email. A member of our team will be in touch with a set of requests.

Please note that your manuscript will not be scheduled for publication until you have made the required changes, so a swift response is appreciated.

IMPORTANT: The editorial review process is now complete. PLOS will only permit corrections to spelling, formatting or significant scientific errors from this point onwards. Requests for major changes, or any which affect the scientific understanding of your work, will cause delays to the publication date of your manuscript.

Should you, your institution's press office or the journal office choose to press release your paper, you will automatically be opted out of early publication. We ask that you notify us now if you or your institution is planning to press release the article. All press must be co-ordinated with PLOS.

Thank you again for supporting Open Access publishing; we are looking forward to publishing your work in PLOS Neglected Tropical Diseases.

Best regards,

Robert Adamu SHEY, Ph.D.

Guest Editor

PLOS Neglected Tropical Diseases

Maria Victoria Periago

Section Editor

PLOS Neglected Tropical Diseases

Shaden Kamhawi

co-Editor-in-Chief

PLOS Neglected Tropical Diseases

orcid.org/0000-0003-4304-636XX

Paul Brindley

co-Editor-in-Chief

PLOS Neglected Tropical Diseases

orcid.org/0000-0003-1765-0002

***********************************************************

Reviewer's Responses to Questions

**Key Review Criteria Required for Acceptance?**

As you describe the new analyses required for acceptance, please consider the following:

**Methods**

-Are the objectives of the study clearly articulated with a clear testable hypothesis stated?

-Is the study design appropriate to address the stated objectives?

-Is the population clearly described and appropriate for the hypothesis being tested?

-Is the sample size sufficient to ensure adequate power to address the hypothesis being tested?

-Were correct statistical analysis used to support conclusions?

-Are there concerns about ethical or regulatory requirements being met?

Reviewer #2: The current version meets the reviewers' recommendation and looks clearer. I have no further comments on the current manuscript. The methodology section is scientifically sound.

**********

**Results**

-Does the analysis presented match the analysis plan?

-Are the results clearly and completely presented?

-Are the figures (Tables, Images) of sufficient quality for clarity?

Reviewer #2: The authors addressed the reviewers' comments in the revised manuscript. The current version is satisfactory.

**********

**Conclusions**

-Are the conclusions supported by the data presented?

-Are the limitations of analysis clearly described?

-Do the authors discuss how these data can be helpful to advance our understanding of the topic under study?

-Is public health relevance addressed?

Reviewer #2: The authors addressed the reviewers' comments in the revised manuscript. The current version is satisfactory.

**********

**Editorial and Data Presentation Modifications?**

Use this section for editorial suggestions as well as relatively minor modifications of existing data that would enhance clarity. If the only modifications needed are minor and/or editorial, you may wish to recommend “Minor Revision” or “Accept”.

Reviewer #2: Accept

**********

**Summary and General Comments**

Use this section to provide overall comments, discuss strengths/weaknesses of the study, novelty, significance, general execution and scholarship. You may also include additional comments for the author, including concerns about dual publication, research ethics, or publication ethics. If requesting major revision, please articulate the new experiments that are needed.

Reviewer #2: The authors addressed the reviewers' comments in the revised manuscript. The current version is satisfactory.

**********

PLOS authors have the option to publish the peer review history of their article (what does this mean?). If published, this will include your full peer review and any attached files.

If you choose “no”, your identity will remain anonymous but your review may still be made public.

**Do you want your identity to be public for this peer review?**  For information about this choice, including consent withdrawal, please see our Privacy Policy.

Reviewer #2: No

# Editor report: Acceptance letter

**DOI:** 10.1371/journal.pntd.0013954.r004

**Authors:** Maria Victoria Periago, Robert Adamu SHEY

Dear Ms. Norman,

We are delighted to inform you that your manuscript, "Assessing diagnostic accuracy of onchocerciasis rapid diagnostic tests using Bayesian latent class models," has been formally accepted for publication in PLOS Neglected Tropical Diseases.

We have now passed your article onto the PLOS Production Department who will complete the rest of the publication process. All authors will receive a confirmation email upon publication.

The corresponding author will soon be receiving a typeset proof for review, to ensure errors have not been introduced during production. Please review the PDF proof of your manuscript carefully, as this is the last chance to correct any scientific or type-setting errors. Please note that major changes, or those which affect the scientific understanding of the work, will likely cause delays to the publication date of your manuscript. Note: Proofs for Front Matter articles (Editorial, Viewpoint, Symposium, Review, etc...) are generated on a different schedule and may not be made available as quickly.

Soon after your final files are uploaded, the early version of your manuscript will be published online unless you opted out of this process. The date of the early version will be your article's publication date. The final article will be published to the same URL, and all versions of the paper will be accessible to readers.

For Research Articles, you will receive an invoice from PLOS for your publication fee after your manuscript has reached the completed accept phase. If you receive an email requesting payment before acceptance or for any other service, this may be a phishing scheme. Learn how to identify phishing emails and protect your accounts at https://explore.plos.org/phishing.

Thank you again for supporting open-access publishing; we are looking forward to publishing your work in PLOS Neglected Tropical Diseases.

Best regards,

Shaden Kamhawi

co-Editor-in-Chief

PLOS Neglected Tropical Diseases

Paul Brindley

co-Editor-in-Chief

PLOS Neglected Tropical Diseases
